# Cell Line Generation: Relying on tricks or tools of the trade?

**DOI:** 10.1186/1753-6561-9-S9-P16

**Published:** 2015-12-14

**Authors:** Jeff JC Hou, Eric Zhu, Michael Song, Kar M Leung, Martina Jones, Trent P Munro, Peter P Gray

**Affiliations:** 1 Australian Institute for Bioengineering and Nanotechnology (AIBN), University of Queensland Corner of Cooper and College Roads, Brisbane, QLD 4067, Australia

## Background

Cell line generation (CLG) in the scope of bioproduction can be defined as a method to isolate a single cell expressing a recombinant protein of interest. The standard method of CLG often involves the introduction of the transgene into a cell in an attempt to use the cellular machinery for transcription, translation and secretion. The use of random genomic integration via auxotrophic selection markers creates different layers of complexity, which leads to significant variations in growth, productivity and stability among the subsequent population[1]. The use of epigenetic modulators with genetically improved cell lines have improved the "quality" of the resistant pools[2], while high throughput technologies have simplified the clonal isolation process[3]. However this "blackbox" approach still requires the need to screen hundreds or thousands of individual cells to find a line with the right quality attributes for manufacturing[4]. The challenge for CLG is to significantly reduce the timeline of this process while ensuring robustness and quality of the subsequent clones[5].

## Materials and methods

The changing landscape of CLG has resulted in the inclusion of robotics and high throughput technologies such as flow cytometry into development pipelines. The standard CLG method may include (but not limited to) transfection, selection and stable pool generation followed by a number of rounds of clonal enrichment using the new technologies to isolate cells with the necessary quality attributes for product manufacturing. However we present a single step method to isolate CHO cells for the expression of monoclonal antibodies (mAb). The method employed here uses semi solid cloning as well as the ClonePix FL (Molecular Devices) to isolate mAb producing CHO cells. The method examines directly seeding transfected cells into a semi-solid matrix for selection, propagation and subsequent isolation. By combining the selection with the isolation in a single step, this direct approach allows for a more efficient process in identifying a "serendipity event", i.e. a single cell that has been transfected with the vector containing the gene of interest, undergone random integration/s at a non-essential locus and now has the ability to express the recombinant protein of interest. Transfected cells were seeded into semi-solid matrix at different seeding densities and then the positive colonies were isolated using the ClonePix FL. The clones were assessed for growth and productivity between the single step and the standard methods.

## Results

To assess the single step method, we examined the growth, productivity and interclonal diversity from the isolated clones. For colony formation in the semi solid matrix, initial seeding density was increased from 500-1000 cells/mL as recommended [2] to 40,000-80,000 cells/mL. Using the Clone Select Imager (Molecular Devices), visible colonies were seen in the semi solid matrix. An initial assessment via the FITC intensity of the in situ fluorescence complex between the Clone Detect (Molecular Devices) and the protein of interest showed a higher signal from the single step method when compared to the standard method. Both methods showed that isolated clones were able to reach 10 million cells/mL with specific productivity ranging from 10 to 50 pg/cell/day. It was clear the standard method provided CHO clones with better growth characteristics while the single step method allowed for the isolation of clones with significantly greater specific productivity.

## Conclusions

The single step method presents a simple change in methodology for increasing the probability of isolating a mAb expressing CHO line without changing the fundamental process. In fact, by directly seeding the transfected cells into the semi solid matrix, the method presents a more robust process eliminating the need for bulk selection and a resistant pool, while also reducing current timelines for CLG. With the rapid changes in the biopharmaceutical industry, especially in biosimilar developments[6], being able to establish a simple and robust process for CLG can have a significant impact on both novel and biosimilar pipelines.

## Acknowledgements

The authors acknowledge the Australian Commonwealth Government's NCRIS Program and co-support from the Queensland State Government.
